# Prevalence estimation by joint use of big data and health survey: a demonstration study using electronic health records in New York city

**DOI:** 10.1186/s12874-020-00956-6

**Published:** 2020-04-06

**Authors:** Ryung S. Kim, Viswanathan Shankar

**Affiliations:** Department of Epidemiology and Population Health, Albert Einstein College of Medicine, 1300 Morris Park Ave, Bronx, NY 10461 USA

**Keywords:** Big data, Electronic health records, Multiple imputations, Measurement error, Selection bias, Population health surveillance

## Abstract

**Background:**

Electronic Health Records (EHR) has been increasingly used as a tool to monitor population health. However, subject-level errors in the records can yield biased estimates of health indicators. There is an urgent need for methods to estimate the prevalence of health indicators using large and real-time EHR while correcting the potential bias.

**Methods:**

We demonstrate joint analyses of EHR and a smaller gold-standard health survey. We first adopted Mosteller’s method that pools two estimators, among which one is potentially biased. It only requires knowing the prevalence estimates from two data sources and their standard errors. Then, we adopted the method of Schenker et al., which uses multiple imputations of subject-level health outcomes that are missing for the subjects in EHR. This procedure requires information to link some subjects between two sources and modeling the mechanism of misclassification in EHR as well as modeling inclusion probabilities to both sources.

**Results:**

In a simulation study, both estimators yielded negligible bias even when EHR was biased. They performed as well as health survey estimator when EHR bias was large and better than health survey estimator when EHR bias was moderate. It may be challenging to model the misclassification mechanism in real data for the subject-level imputation estimator. We illustrated the methods analyzing six health indicators from 2013 to 14 NYC HANES and the 2013 NYC Macroscope, and a study that linked some subjects in both data sources.

**Conclusions:**

When a small gold-standard health survey exists, it can serve as a safeguard against potential bias in EHR through the joint analysis of the two sources.

## Background

Electronic Health Records (EHR) has been increasingly used as a tool for public health surveillance by local and national jurisdictions [1]. For example, recent studies in New York City (NYC) reported that the prevalence estimates from NYC Macroscope, an EHR-based surveillance system in NYC [2], were comparable to the survey-based estimates for diabetes, hypertension, and smoking [3, 4]. EHR often cover more people (*n* ≥ 100,000) than traditional population health surveys and, and once the infrastructure is in place, the data collection occurs in near real-time without additional recruitment or interviewing cost.

Despite these advantages, the prevalence estimates from EHR can often be biased mainly due to two causes. The first is selection bias. That is, EHR may not represent the target population. For example, the patient population from NYC Macroscope under-represents young men, over-represents patients living in high poverty neighborhoods. It only includes patients who visit primary care doctors connected to a particular EHR system [2]. The selection bias can be corrected, if modeled correctly, by post-stratification. The other source of error is the misclassifications of health outcomes, which is the main interest of our study. It comprises measurement error (e.g., due to the use of non-standardized instruments across sites), extraction error, or the collection of proxy-measurement (e.g., due to the recording without distinction of both self-report and actual measurements). McVeigh et al. [3] reported such subject-level discrepancies by examining a chart review of participants who both visited NYC Macroscope providers and also participated in the NYC Health and Nutrition Examination Survey (HANES), a population-representative survey with field interviews and biospecimen collection. Assuming NYC HANES measurements as “gold-standard,” the chart review found a 5% subject-level error for obesity, 19% for depression, and 19% for influenza vaccination. Notably, the sensitivity (i.e., the proportion of the medical condition identified in NYC HANES also indicated in the EHR) was as low as 31% for depression and 19% for influenza vaccination. In a later study, McVeigh et al. [5] extracted chart data from more than 20 additional EHR software systems from primary care providers and repeated similar study for 190 participants of the 2013–14 NYC HANES. For the public health surveillance system using EHR records, there is an urgent need for methods to estimate the prevalence of health indicators using large and real-time EHR while correcting the potential bias using external sources.

Many existing methods allow investigators to pool multiple data sources and some may be suitable for the unique context of combining big data with a small gold-standard survey. They can be classified by whether the subjects are linked at the individual level and whether potential biases are accounted for. For data sources that are unavailable at the individual level, aggregate statistics are pooled from the sources. For example, Thompson [6] developed methods to combine aggregate statistics from standardized surveys by an international tobacco control project to find programs that are effective in reducing tobacco use. She studied several approaches including a model with random effects for the country. However, her model assumed that all surveys were equally likely to be biased and the bias across countries canceled each other out. There are a handful of works that account for pooling a gold-standard source with potentially biased sources [7–11]. Earlier, Mosteller [9] studied ways to combine the means from two samples when one is potentially biased. Mosteller’s estimator, chosen as one end of the methods, will be discussed further in the following section. Lohr and Brick [7] explored methods for pooling domain-level estimates from two surveys that measure victimization prevalence: their gold-standard survey, the United States National Crime Victimization Survey, and a larger but potentially biased telephone companion survey. In their study, they compared ten methods that combine a gold-standard survey with another biased data source. The methods included calibration methods, weighted averages of the estimators from the two sources without any bias adjustment (i.e. unadjusted dual-frame estimators), with bias adjustment pooled across the domains, and with domain-specific bias adjustment. The last method performed the best. Another estimator that performed well was the multiplicative bias estimator published earlier [11]. Manzi et al. [8] used a Bayesian hierarchical model to pool domain-level smoking prevalence estimates from seven surveys in the eastern regions of England. Similarly, Raghunathan et al. [10] used Bayesian hierarchical model to combine a potentially biased county-level prevalence of cancer outcomes and risk factors from a larger telephone survey, The Behavioral Risk Factor Surveillance System, with an unbiased (or less biased) face-to-face National Health Interview Survey (NHIS) covering fewer counties and fewer households.

When data are available at the individual level, Kim and Rao [12] developed a method to combine a small survey with outcome measurement and auxiliary information with a larger independent survey with only auxiliary information. Park et al. [13] developed a model to pool one gold-standard source with outcome measurement and auxiliary information with another independent source with a potentially biased outcome and the same auxiliary measure. Schenker et al. [14] used multiple imputations to combine self-reported outcomes from a large survey, NHIS, with a smaller NHANES that has both clinical and self-report outcomes. They imputed clinical measurement of health outcomes for the participants of the larger survey by modeling both the underlying mechanism of misspecification of outcomes and the mechanism of inclusion to each survey. We will study further this method in the following section as another end of the methods. For more than two proxy outcome variables measured with lagged overlaps, Gelman et al. [15] and He et al. [16] used similar multiple imputation approaches.

In this study, we aim to demonstrate that the joint analysis of a large EHR with a much smaller gold-standard health survey can improve the accuracy of the prevalence estimates. Our aim is not to study all available methods but instead to demonstrate two statistical procedures at both ends of spectrum. First, we adopt Mosteller’s method [9] to pool two estimators when one is potentially biased. It only requires knowing the prevalence estimates from two data sources and their standard errors. Second, we adopt the method of Schenker et al. [14], which uses iterative multiple imputations of subject-level health outcomes for both surveys. This procedure requires information to link some subjects between two sources and modeling the mechanisms underlying the misclassifications in EHR as well as modeling inclusion probabilities to both sources. We demonstrate the statistical properties of the two estimators using simulation studies. Finally, we illustrate these methods analyzing 2013–14 NYC HANES and the 2013 NYC Macroscope and a small study that linked some subjects between the two sources.

## Methods

**Fig. 1:**
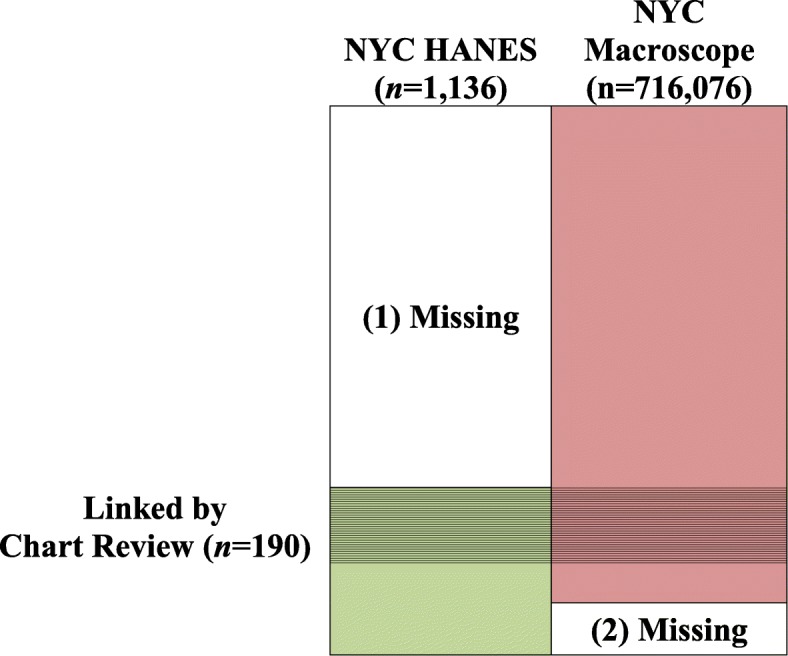
Data elements in the 2013–14 NYC HANES, limited to the in-care population and stratified by whether the participant was in the chart review study, and 2013 NYC Macroscope

We consider two data sources. First is a health survey of a smaller sample *S*_1_ with survey weights *w*_1_ that is representative of the target population. Measurement *Y*_1_ in the survey is the gold-standard and hence $$ {\hat{p}}_1 $$$$ ={\sum}_{i\in {S}_1}{w}_{1,i}{Y}_{1,i}/\sum {w}_{1,i} $$ is an unbiased estimator of the prevalence of interest *p*_1_. Another data source is EHR of a larger sample *S*_2_ that becomes representative of the population with post-stratified weights *w*_2_. Measurement *Y*_2_ in the EHR may have subject-level errors and $$ {\hat{p}}_2 $$$$ ={\sum}_{i\in {S}_2}{w}_{2,i}{Y}_{2,i}/\sum {w}_{2,i} $$ may be a biased estimator of *p*_1_. We denote logit of the prevalence as *ϕ*_1_ =logit(*p*_1_) and logit of prevalence estimators from the two sources as *y*_1_ =logit($$ {\hat{p}}_1 $$) and *y*_2_ =logit($$ {\hat{p}}_2 $$). We assume that the covariance between two estimators is ignorable since the number of the overlapping subjects (*S*_1_∩*S*_2_) is typically very small relative to the size of EHR (*S*_2_). We can link the subset of the overlapping subjects (*S*_c_) between the two sources. Figure 1 outlines the data structure. We used statistical software R for all statistical analyses [17, 18].

### Mosteller estimator

At the core of the problem is a simple question: “Can we gain by pooling two estimates when one is possibly biased but from a larger sample?” Earlier, Mosteller (1948) [9] studied whether to pool two sample means when one is potentially biased. He compared the mean squared error (MSE) of various mean estimators: the unbiased mean, test-then-pool estimator (i.e., pooling two means only when the mean difference was not significant), and maximum likelihood estimator (MLE) assuming mean-zero Gaussian bias. The last estimator showed the least MSE. We adopt his approach to account for unequal sample sizes and unequal variances. The estimator is a weighted average of *y*_1_ and *y*_2_:

$$ {\hat{\phi}}^{\mathrm{M}}=\left({k}_1{y}_1+{k}_2{y}_2\right)/\left({k}_1+{k}_2\right). $$

It can be shown that the MSE of this family of estimators is minimized when ${k}_1=1/{\upsigma}_1^2$, ${k}_2=1/\left({\uptau}^2+{\upsigma}_2^2\right)$, where σ_1_ and σ_2_ are the standard errors of *y*_1_ and *y*_2_, and τ = *E*(*y*_2_) − *ϕ*_1_ is the bias of *y*_2_. The estimator is also the MLE of *ϕ*_1_ under the model *y*_*j*_ = *ϕ*_1_ + 1(j = 2)*θ* + *e*_*j*_ where *θ* and *e*_*j*_ are mutually independent zero-mean normal variable with variance *τ*^2^ and ${\sigma}_j^2$, respectively. The variance and bias parameters were estimated by consistent estimators ${\hat{\upsigma}}_1^2={s}_1^2$, ${\hat{\upsigma}}_2^2={s}_2^2$ and ${\hat{\uptau}}^2={\left({y}_1-{y}_2\right)}^2.$ For example, ${s}_j^2$ can be the sample variance estimated using survey weights.

The same estimator can also be derived from an approximate Bayesian perspective [19] by setting a prior to the asymptotically normal sampling distribution of *y*_*j*_. If we set a non-informative prior (i.e. normal with infinite variance) of *ϕ*_1_, and zero-mean normal prior of the bias *E*(*y*_2_) − *ϕ*_1_ with variance *τ*^*2*^, then the posterior distribution of *ϕ*_1_ can be shown to be normal with mean ${\hat{\phi}}^{\mathrm{M}}$ and variance ${\sigma}_1^2\left({\sigma}_2^2+{\tau}^2\right)/\left({\sigma}_1^2+{\sigma}_2^2+{\tau}^2\right)$. τ measures the prior belief in closeness of the prevalence measured by EHR and health survey. The 95% highest density credibility interval of the logit prevalence is given as

$$ {\hat{\phi}}^{\mathrm{M}}\pm 1.96\ {\sigma}_1\sqrt{\left({\sigma}_2^2+{\tau}^2\right)/\left({\sigma}_1^2+{\sigma}_2^2+{\tau}^2\right)} $$

The estimator, while less efficient than the subject-level imputation estimator below, is simpler to implement by practitioners who often do not have resources to link subjects in two sources or model the mechanisms of the misclassifications in EHR.

### Subject-level imputation estimator

#### Misclassification model

We adapted the approach by Schenker et al. [14] and modeled the misclassification between the binary outcomes of *i*^th^ subject in health survey (*Y*_1, *i*_) and EHR (*Y*_2, *i*_):

1 $$ \mathrm{logit}\ P\left({Y}_{2,i}=1|{Y}_{1,i}={y}_{1,i}\right)={\beta}_{0l}+{\beta}_{1l}{z}_i+{\beta}_{2l}{y}_{1,i} $$

2 $$ \mathrm{logit}\ P\left({Y}_{1,i}=1|{Y}_{2,i}={y}_{2,i}\right)={\gamma}_{0l}+{\gamma}_{1l}{z}_i+{\gamma}_{2l}{y}_{2,i} $$

where *z*_i_ is a vector predictor. Since the relationship may depend on the design factors of surveys, the model is stratified by four levels (*l* = 1, 2, 3, 4) divided by the quartiles of the inclusion probabilities to the health survey as *q*_*11*_, *q*_*12*_, *q*_*13*_ and to the EHR as *q*_*21*_, *q*_*22*_, *q*_*23*_.

#### Model for inclusion to each source

Since the inclusion probabilities to health survey (*π*_1i_) are unknown for most EHR subjects, we model them by a model, logit *π*_1i_ = *a*_0+_*a*_1_*u*_*i*_, where *u*_i_ is a vector of survey design factors. The model is fit over entire EHR subjects weighted by their post-stratified weights (*w*_2_). Similarly, we model the inclusion probability to EHR logit *π*_2i_ = *b*_0+_*b*_1_*v*_*i*_ and fit it over entire health survey subjects weighted by their survey weights.

#### Bayesian iterative regression imputation

While we are ultimately interested in imputing missing health survey outcomes (1) in Fig. 1, we follow Schenker et al. [14] and perform iterative imputations between two models M1, to impute missing EHR values (2) in the figure, and M2, to impute missing health survey values (1) in the figure. This is repeated B times. Imputing missing EHR values (2) in the figure increases sample size when fitting M2, the model we are ultimately interested. The additional variation caused by using imputed values was accounted for by the multiple imputation standard error formula below. The following is the detailed procedure.

To impute missing *Y*_2,i_, we divided the subjects *S*_*1*_ ∪ *S*_*2*_ into 4 (*l* = 1, …, 4) groups by the quartiles *q*_*21*_, *q*_*22*_, *q*_*23*_, and within each group fit Bayesian regression model M1 with a weakly informative prior for *β*_*l*_ = (*β*_*0l,*_*β*_*1l,*_*β*_*2l*_) of independent Cauchy distributions with 2.5 scale and zero location, first on the subjects *S*_*c*_ whose identities can be linked between two data sources. Then, we drew a posterior sample of *β*_*l*_, and in turn *Y*_2,i_ conditional on *β*_*l*_ for all health survey subjects missing *Y*_2,i_. Subsequently, treating this imputed *Y*_2,i_ as observed, we imputed missing *Y*_1,*i*_ by dividing the subjects into 4 groups by *q*_*11*_, *q*_*12*_, *q*_*13*_ and fitting the regression model M2 on all EHR subjects with independent Cauchy prior for *γ*_*l*_ = (*γ*_*0l*_*, γ*_*1l*_*, γ*_*2l*_) with 2.5 scale and zero location. We drew a posterior sample from *γ*_*l*_, then in turn *Y*_1,i_ for all EHR subjects missing *Y*_1,i_. We iterated *B* times to fit models M1 and M2, treating imputed values from the previous step as observed and imputing the missing outcome variables until convergence. Then we calculated a prevalence estimator ${\hat{p}}_m$ = ${\sum}_{i\in {S}_2}{w_2}_i{\hat{Y}}_{m,1,i}/\sum {w_2}_i$ based on the imputed health survey measurements of all EHR subjects. Notice that the outcome values were imputed only when they are missing. In other words, ${\hat{Y}}_{m,1,i}$ = *Y*_1, *i*_ for subjects whose health survey outcome was observed. Finally, we combined inferences from *M* such multiple imputations. The resulting prevalence estimator is unbiased when the specified models are correct:

$$ {\hat{P}}^{\mathrm{R}}={\sum}_{m=1}^M{\hat{p}}_m/M $$

The standard error of ${\hat{\phi}}^{\mathrm{R}}$ =logit(${\hat{P}}^{\mathrm{R}}$) was estimated by the standard way [20, 21]:

$$ SE\left({\hat{\phi}}^{\mathrm{R}}\right)=\sqrt{W+\left(1+1/M\right)B} $$

where *W*= ${\sum}_m{s}_m^2/M$, *B*= ${\sum}_m{\left({\hat{\phi}}_m-{\hat{\phi}}^{\mathrm{R}}\right)}^2/\left(M-1\right)$, and *s*_*m*_ is the naïve standard error of the logit prevalence (${\hat{\phi}}_m$) calculated from *m*^th^ imputation. Since the overlap between two sources can be small, we used Barnard-Rubin degrees of freedom [22, 23] to compute credibility intervals, first in log-odds scale before they were transformed to probability scale.

## Results

### Simulation studies

We performed simulation studies to assess the performance of the methods under various settings. We generated correlated binary outcomes (Y_1_, Y_2_) of a target population (*N* = 10,000,000) whose conditional distributions follow logistic models: logit P(Y_1_ = 1|Y_2_) = *η*_10_+ *φ* Y_2_ and logit P(Y_2_ = 1|Y_1_) = *η*_01_+ *φ* Y_1_ where *η*_10_ = *γ*_0_+ *γ*_1_*x*_1_+ *γ*_2_*x*_2_, *η*_01_ = *β*_0_+ *β*_1_*x*_1_+ *β*_2_*x*_2_. To do so, we first generated an independent Bernoulli variable *x*_1_ with success probability .5 and a standard normal variable *x*_2_. Then we generated the correlated binary outcomes (Y_1_, Y_2_) which has 4 possible outcomes (0,0) (0,1), (1,0), (1,1) with corresponding joint probabilities *p*_00_, *p*_01_, *p*_10_, *p*_11_ where *p*_11_: *p*_10_:*p*_01_: *p*_00_ = exp.(*φ + η*_10_ *+ η*_01_): exp.(*η*_10_):exp.(*η*_01_):1. This set up guarantees that the conditional distributions of outcomes are the two stated logistic models. The log odds ratio *φ* and the linear coefficients were set so that the true prevalence based on two datasets were *p*_1_ = *p*_11_+ *p*_10_ = 0.3 and *p*_2_ = *p*_11_+ *p*_01_ = 0.3, 0.31, 0.32, 0.33, or 0.35.

Then, we randomly selected subjects for the health survey (*n*_1_ = 250, 500, or 1000) and EHR (*n*_2_ = 100,000) by inclusion probabilities proportional to logit *π*_1i_ = *a*_0_ + *a*_1_*u*_1*i*_ + *a*_2_*u*_2*i*_ + *a*_3_*x*_1*i*_ and logit *π*_2i_ = *b*_0_ + *b*_1_*u*_1*i*_ + *b*_2_*u*_2*i*_ + *b*_3_*x*_1*i*_. *u*_1_ was an independent Bernoulli variable with success probability .5 and *u*_2_ was a standard normal variable. We set (*a*_0_, *a*_1_*, a*_2_*, a*_3_) = (*b*_0_, *b*_1_*, b*_2_*, b*_3_) = (1,1,1, 0.187). *x*_1_, the predictor of misclassification, was also included as a survey design factor so that the missing mechanism is missing-at-random but not missing-completlely-at-random. Then, we selected more EHR subjects among the health survey participants so that the proportion of health survey participants that are also in EHR is 20, 50%, or 100%. Finally, we deleted the values of *Y*_1_ and *π*_1_ for the subjects not in the health survey and *Y*_2_ for the subjects not in EHR. All *π*_2_ values were deleted as inclusion probabilities are unknown in typical EHR.

For each simulated survey and EHR, we used *u*_1_, *u*_2_, and *x*_1_ to calculate post-stratified weights *w*_2_ for the EHR. Then we calculated four prevalence estimates: estimator based only on the health survey, estimator based only on EHR, Mosteller estimator, and the subject-level imputation estimator. For the subject-level imputation estimator, we included burn-in iterations and combined inferences of *M* = 30 multiple imputations. The overall process of the generation of the target population, sampling health survey and EHR from the population, and calculating the prevalence estimates was repeated 200 times.

Table 1 shows the average prevalence estimates by the four estimators. The size of the health survey (*n*_1_) and the size of subjects linked between two sources (*n*_12_) were both 500. Health survey estimator was unbiased in all settings. On the contrary, EHR estimator was biased except when there was no misclassification bias (i.e., *p*_2_ = 0.3), in which case post-stratification successfully adjusted for the selection bias. Both Mosteller estimator and the subject-level imputation estimator showed less than 3% bias in all settings.

**Table 1:** Simulation studies: prevalence estimate by four methods

True Population Prevalence		Prevalence Estimate (95% CI)			
Prevalence (*p*_1_) based on outcome in health survey (*Y*_1_)	Prevalence (*p*_2_) based on outcome in EHR (*Y*_2_)	Health Survey (*n*_1_ = 500)	Post-stratified EHR	Mosteller estimator	Subject-level imputation estimator
0.3	0.30	0.300	0.299	0.300	0.303
0.3	0.31	0.300	0.309	0.303	0.302
0.3	0.32	0.299	0.319	0.305	0.302
0.3	0.33	0.298	0.329	0.305	0.303
0.3	0.35	0.300	0.349	0.308	0.304

The size of health survey (*n*_1_) and the size of subjects linked between two sources (*n*_12_) are both 500

Table 2 shows the MSE of the estimators. When bias was less than or equal to 5% (i.e., *p*_2_ = 0.3 or 0.31), the EHR estimator outperformed the health survey estimator due to a larger sample size. When the bias was more substantial, however, it overwhelmed the benefit from the sample size. Then, the subject-level imputation model and the Mosteller estimator performed better than the estimators based only on either source. Notably, they either outperformed or were similar to the health survey estimator in all settings. Between the two, the Mosteller estimator performed better than the subject-level imputation estimator when bias was small to moderate (*p*_2_ = 0.3–0.33), but worse when bias was large (*p*_2_ = 0.35).

**Table 2:** Simulation studies: square root of MSE of four methods

True Population Prevalence		Squared Root of MSE			
Prevalence (*p*_1_) based on outcome used in health survey (*Y*_1_)	Prevalence (*p*_2_) based on outcome used in EHR (*Y*_2_)	Health Survey (*n*_1_ = 500)	Post-stratified EHR	Mosteller estimator	Subject-level imputation model
0.3	0.30	0.021	**0.002**	0.015	0.019
0.3	0.31	0.021	**0.009**	0.017	0.019
0.3	0.32	0.022	0.019	**0.018**	0.021
0.3	0.33	0.021	0.029	**0.020**	0.021
0.3	0.35	**0.021**	0.049	0.023	**0.021**

Square root of MSE for estimating *p*_1_ is shown. The size of health survey (*n*_1_) and the size of subjects linked between two sources (*n*_12_) are both 500. For each row, the best performing method in each row is highlighted in bold

We studied how the size of the health survey and the size of subjects linked between two sources affect the performance (Table 3). We fixed the true prevalence (*p*_1_) at 0.3 and the prevalence (*p*_2_) measured from EHR (*Y*_2_) at 0.32. The EHR estimator performed best when the health survey was small (*n*_1_ = 250) but Mosteller’s estimator performed best when the health survey size was moderate (*n*_1_ = 500, 1000). The subject-level imputation estimator requires enough size of subjects linked between two sources. Mostellers’ method, on the other hand, performed well in most settings.

**Table 3:** Simulation studies: square root of MSE by different sample sizes

Size of health Survey (*n*_1_)	Size of subjects linked between two sources (*n*_12_)	Health Survey	Post-stratified EHR	Mosteller estimator	Subject-level imputation model
250	50	0.033	**0.019**	0.026	0.049
	125	0.031	**0.019**	0.024	0.046
	250	0.030	**0.019**	0.023	0.040
500	100	0.022	0.019	**0.019**	0.032
	250	0.023	0.019	**0.019**	0.031
	500	0.022	0.019	**0.018**	0.021
1000	200	0.016	0.019	**0.014**	0.027
	500	0.015	0.019	**0.015**	0.022
	1000	0.016	0.019	0.015	**0.014**

Prevalence (*p*_1_) measured in health survey (*Y*_1_) is fixed at 0.3 and the prevalence (*p*_2_) measured in EHR (*Y*_2_) is fixed at 0.32. The size of EHR (*n*_2_) is fixed at 100,000. Square root of MSE for estimating *p*_1_ is shown. The best performing method in each row is highlighted in bold

### Analysis of NYC macroscope and NYC HANES

We illustrate the methods with data from NYC. To protect patient privacy, the authors did not directly access the data but submitted R codes to the NYC Department of Health and Mental Hygiene (DOHMH) and received back the results of the joint analysis of two data sources presented below.

#### Description of data sources

NYC Macroscope is an EHR-based surveillance system developed by the NYC DOHMH in collaboration with the City University of New York School of Public Health to estimate the prevalence of chronic diseases and risk factors for adult population (20 years or older) in care by participating primary care providers in NYC [2, 5]. The data were available only as aggregate data stratified by age group, sex, and neighborhood poverty level. Detailed provider and patient inclusion and exclusion criteria are documented elsewhere [2]. In this study, we used the 2013 data that included 716,076 patients.

The 2013–14 NYC HANES is a population-representative survey of NYC residents aged 20 or older (*n* = 1527) with the interview, physical examination and biospecimen collection [24]. The data used in this study were limited to in-care participants (i.e., participants who have seen a provider for primary care in the previous year; *n* = 1135). Recently, a chart review study was conducted among a subsample (*n* = 190) of in-care participants from NYC HANES (Fig. 1) [5]. In their study, more than 20 EHR from primary care providers were abstracted for each chart review participant, and the data were linked to the NYC HANES data at the individual level. The chart review sample consisted of participants who received primary care from NYC Macroscope or non-NYC Macroscope providers. Because there was little difference in demographic and clinical characteristics between the two groups, we used data from all participants in this study. They performed the chart review on subjects enrolled in NYC HANES 2013–14 (*n* = 1524) who had doctors visit during the year (*n* = 1135) and signed a consent form and Health Insurance Portability & Accountability Act (HIPPA) waiver (*n* = 491) whose EHR were available and valid (*n* = 190).

#### Definition of health indicators

We selected six health indicators in the sources to demonstrate the methods: hypertension diagnosis, diabetes diagnosis, smoking, obesity, depression, and influenza vaccination. Newton-Dame and her collegues describes these indicators in detail [2]. Hypertension diagnosis was defined as either systolic blood pressure ≥ 140 mmHg or diastolic blood pressure ≥ 90 mmHg or an existing record of hypertension diagnosis (based on ICD-9 in NYC Macroscope and self-report in NYC HANES). Diabetes indicator was based on the presence of an ICD-9 diagnosis in NYC Macroscope and self-report in NYC HANES. Smoking was based on an indication of ‘current smoking’ in the most recent smoking status in the NYC Macroscope and based on a self-report of current smoking in NYC HANES. The obesity indicator was based on the most recent body mass index (BMI) ≥ 30 in NYC Macroscope and based on the measured height and weight at the interview in NYC HANES. Depression indicator was based on the presence of an ICD-9 depression diagnosis ever recorded, or a Patient Health Questionnaire (PHQ-9) score ≥ 10 in NYC Macroscope and based on a self-reported diagnosis or a PHQ-9 score ≥ 10 at interview in NYC HANES. Influenza vaccination indicator was based on the presence of a relevant ICD-9/CPT/CVX code in NYC Macroscope and based on the self-report of receiving influenza vaccination in the past 12 months in NYC HANES.

#### Illustration of the methods on NYC data

The NYC Macroscope used post-stratification to address the selection bias of Macroscope data [25, 26] by matching the joint distribution of gender, age group, and neighborhood-level poverty to that of the city’s in-care population. The prevalence estimates among the in-care city population-based on NYC HANES and NYC Macroscope were close for hypertension diagnosis (NYC HANES 34.3% vs. NYC Macroscope 33.7%), moderately different for diabetes diagnosis (13.3% vs 14.8%), smoking (17.3% vs. 15.9%), and obesity (31.7% vs. 29.1%), and significantly different for depression (19.0% vs. 8.6%) and influenza vaccination (48.6% vs. 21.2%). The discrepancies in the depression prevalence and influenza vaccination rate were likely due to the under-diagnosis of depression in primary care settings and influenza vaccination outside of clinics (e.g., pharmacies) that are not recorded by the primary care EHR. The population characteristics in NYC HANES and NYC Macroscope for the adult in-care population are described elsewhere [27].

We estimated prevalence by the four estimators: estimator based only on NYC HANES, estimator based only on Macroscope data, Mosteller estimator, and the subject-level imputation estimator. We assumed that NYC HANES was the gold standard since data were collected using a population-representative sample design with a controlled and standardized data collection. The chart review study with 190 subjects whose identities were linked between NYC HANES and NYC Macroscope enabled us to calculate the subject-level imputation estimates for which we used age group, sex, and neighborhood poverty level as covariates for inclusion models and misclassification models. There was a lack of predictors that could properly model misclassifications in the EHR, such as hospital size, instrument labels, or types of visits.

Mosteller prevalence estimates showed improvement over both NYC HANES and NYC Macroscope estimates (Table 4). In all six health outcomes, they showed smaller standard errors compared to NYC HANES estimates and smaller biases compared to Macroscope estimator. The bias reduction was especially substantial (> 99% reduction) in depression and influenza vaccination estimates because, for these indicators, EHR estimates were given little weight (Table 5). On the other hand, the subject-level imputation estimates did not outperform NYC HANES estimates: their credibility intervals were larger than NYC HANES estimates. This was due to the lack of predictors, as mentioned above, that could model the mechanism of misclassification in EHR. The subject-level imputation method requires us to correctly model the misclassification as well as to approximate the inclusion probabilities to the health survey for the EHR subjects.

**Table 4:** Prevalence estimate and 95% confidence/credibility intervals of select health outcomes among adults in care in New York City (NYC), obtained from the NYC Macroscope 2013 and NYC HANES 2013–14

Outcomes	Prevalence Estimate (95% CI)				
	NYC HANES (*n* = 1135)	Crude NYC Macroscope (*n* = 716,076)	Post-stratified NYC Macroscope	Subject-level imputation model	Mosteller estimator
Hypertension Diagnosis	34.3	33.7	34.7	35.6	34.7
	(31.3, 37.4)	(33.6, 33.8)	(34.6, 34.8)	(30.4, 41.1)	(34.0, 35.4)
Diabetes Diagnosis	13.3	14.8	14.9	13.8	13.9
	(11.3, 15.6)	(15.8, 16.0)	(14.9, 15.0)	(10.6, 17.7)	(11.5, 16.5)
Smoking	17.3	15.9	15.0	19.0	16.9
	(15.1, 19.9)	(15.8, 16.0)	(14.9, 15.1)	(16.0, 22.5)	(14.4, 19.7)
Obesity	31.7	29.1	28.0	30.9	31.1
	(28.7, 34.8)	(29.0, 29.2)	(27.9, 28.1)	(26.5, 35.7)	(27.9, 34.6)
Depression	19.0	8.6	8.3	20.3	18.9
	(16.6, 21.6)	(8.5, 8.6)	(8.3, 8.4)	(17.2, 23.9)	(16.5, 21.5)
Influenza Vaccination	48.6	21.2	21.7	48.2	48.5
	(45.4, 51.8)	(21.1, 21.3)	(21.6, 21.8)	(43.8, 52.5)	(45.3, 51.7)

The units are in percentage

**Table 5:** Relative weights used in Mosteller estimator

Outcomes	NYC HANES:Macroscope
Hypertension Diagnosis	0.075:0.925
Diabetes Diagnosis	0.665:0.335
Smoking	0.812:0.188
Obesity	0.855:0.145
Depression	0.993:0.007
Influenza Vaccination	0.997:0.003

Table 4 also demonstrates that the selection bias in Macroscope was less than the bias due to subject-level misclassifications: the range of differences in prevalence estimates between Macroscope and NYC HANES for diabetes, smoking, and obesity were similar with (1.6–3.7%) and without (1.5–2.6%) post-stratification. However, it decreased to 0.4–0.6% for the Mosteller estimator. The range of differences in depression prevalence and influenza vaccination rate were also similar with (10.7–26.9%) and without (10.4–27.4%) post-stratification but it reduces dramatically to 0.1% for the Mosteller estimator. This shows that post-stratification alone was insufficient to correct the bias in the EHR for these outcomes. But Mosteller estimator and subject-level imputation estimator both used NYC HANES as a safeguard against potential bias in EHR.

## Discussion

Compared to traditional health surveys, EHR has a much larger sample size and the potential to reduce standard errors of prevalence estimates. It can be very helpful in estimating prevalence in small sub-groups of the populations. In NYC Macroscope and our simulation study, we found that the correction of the subject-level error of EHR is necessary and possible.

In the simulation study, the health survey estimator was unbiased, but the standard error was the largest. On the contrary, the bias in EHR estimator can overwhelm the benefit of its sample size. When that happened, both Mosteller estimator and the subject-level imputation estimator yielded negligible bias and small standard errors: they either outperformed or were comparable to the estimators based solely on either source. The subject-level imputation estimator may outperform Mosteller estimator when EHR bias is large. However, the estimator requires enough size of subjects linked between two sources and correctly modeling the mechanism of misclassification as well as modeling inclusion probabilities to both sources.

The difficulty of such a task was demonstrated in the analysis of the NYC data. Mosteller estimators showed considerably smaller standard error than NYC HANES estimates especially when the NYC Macroscope estimates and NYC HANES estimates were close. The subject-level imputation estimator did not outperform NYC HANES estimator in part due to a lack of predictors for misclassification. The predictors for misclassification can be both patient-level characteristics, such as types of visit, and institution-level predictors, such as hospital size or instrument labels. These variables are typically going to be found in EHR (or administrative data sets that accompany EHR), while some patient characteristics will still be found in a health survey. In practice, the fit of the misclassification model should guide the choice between considered approaches, whether to model the underlying mechanism of misclassification or to use Mosteller’s estimator. This can be done, for example, by cross-validated estimation of area under the curve of the receiver operating characteristic (ROC) curve as one moves the probability cutoff in the logistic regression model M2.

In this article, we considered the health survey as the gold standard. Here we acknowledge that survey measurements are rarely unbiased. However, it is often helpful to treat one survey as gold-standard over another. For example, investigators have treated a smaller in-person survey as gold-standard over a larger telephone survey [10], or clinical surveys as gold-standard over self-reported outcomes [14, 28]. EHR are often administrative data collected for billing purposes with non-standardized instruments and protocols, with complex unknown inclusion mechanisms. NYC HANES was designed for health survey purposes by standardized instruments and protocols and collected by representative probability sampling. We assumed that typical bias treatment for the health survey, such as post-stratification and calibration for non-response bias has been successfully performed.

## Conclusions

We demonstrated that the joint use of a small gold-standard health survey with a larger EHR improves accuracy in prevalence estimation. Depending on the available data, one can aim to model the misclassification completely or calculate the weighted average of the prevalence estimates from two sources. The studied approaches can improve the quality of EHR as a public health surveillance tool. In another work, we are extending the methods to model subgroup level prevalence estimators from health surveys and EHR.

## Data Availability

This study includes a secondary analysis of two data sources that are not owned by the authors. Readers can inquire about data by visiting the NYC HANES Project (http://nychanes.org) or contacting NYC DOHMH.

## Abbreviations

BMI: Body mass index
CPT: Common procedural technology
CVX: Vaccine administered
DOHMH: Department of Health and Mental Hygiene
EHR: Electronic health records
ICD-9: International classification of diseases - ninth revision
MLE: Maximum likelihood estimator
MSE: Mean squared error
NHIS: National health interview survey
NYC: New York city
NYC HANES: New York City health and nutrition examination survey
PHQ-9: Patient health questionnaire-9

## References

[1] Paul MM, Greene CM, Newton-Dame R, Thorpe LE, Perlman SE, McVeigh KH (2015). The state of population health surveillance using electronic health records: a narrative review. Popul Health Manag.

[2] Newton-Dame R, McVeigh KH, Schreibstein L, Perlman S, Lurie-Moroni E, Jacobson L (2016). Design of the New York City Macroscope: innovations in population health surveillance using electronic health records. EGEMS (Washington, DC).

[3] Thorpe LE, McVeigh KH, Perlman S, Chan PY, Bartley K, Schreibstein L (2016). Monitoring prevalence, treatment, and control of metabolic conditions in New York City adults using 2013 primary care electronic health records: a surveillance validation study. EGEMS (Washington, DC).

[4] McVeigh KH, Newton-Dame R, Chan PY, Thorpe LE, Schreibstein L, Tatem KS (2016). Can electronic health records be used for population health surveillance? Validating population health metrics against established survey data. EGEMS (Washington, DC).

[5] McVeigh KH, Lurie-Moroni E, Chan PY, Newton-Dame R, Schreibstein L, Tatem KS (2017). Generalizability of indicators from the New York city macroscope electronic health record surveillance system to systems based on other EHR platforms. EGEMS (Washington, DC).

[6] Thompson ME (2008). International surveys: motives and methodologies. Surv Methodol.

[7] Lohr SL, Brick JM (2012). Blending domain estimates from two victimization surveys with possible bias. Can J Stat.

[8] Manzi G, Spiegelhalter DJ, Turner RM, Flowers J, Thompson SG (2011). Modelling bias in combining small area prevalence estimates from multiple surveys. J Royal Stat Soc Ser A.

[9] Mosteller F (1948). On pooling data. J Am Stat Assoc.

[10] Raghunathan TE, Xie D, Schenker N, Parsons VL, Davis WW, Dodd KW (2007). Combining information from two surveys to estimate county-level prevalence rates of cancer risk factors and screening. J Am Stat Assoc.

[11] Ybarra LMR, Lohr SL (2008). Small area estimation when auxiliary information is measured with error. Biometrika.

[12] Kim J, Rao J (2012). Combining data from two independent surveys: a model-assisted approach. Biometrika.

[13] Park S, Kim JK, Stukel D (2017). A measurement error model for survey data integration: combining information from two surveys. Metron.

[14] Schenker N, Raghunathan TE, Bondarenko I (2010). Improving on analyses of self-reported data in a large-scale health survey by using information from an examination-based survey. Stat Med.

[15] Gelman A, King G, Liu C (1998). Not asked and not answered: multiple imputation for multiple surveys: rejoinder. J Am Stat Assoc.

[16] He Y, Landrum MB, Zaslavsky AM (2014). Combining information from two data sources with misreporting and incompleteness to assess hospice-use among cancer patients: a multiple imputation approach. Stat Med.

[17] Gelman A, Su Y (2011). Arm : data analysis using regression and multilevel/hierarchical models.

[18] R Core Team (2016). R: a language and environment for statistical computing.

[19] Wang Z, Kim JK, Yang S (2017). Approximate Bayesian inference under informative sampling. Biometrika.

[20] Gelman A, Hill J (2006). Data analysis using regression and multilevel/hierarchical models.

[21] Rubin DB. Multiple imputation for nonresponse in surveys. New York: Wiley; 2006.

[22] Barnard J, Rubin DB (1999). Small-sample degrees of freedom with multiple imputation. Biometrika.

[23] van Buuren S, Groothuis-Oudshoorn K (2011). Mice: multivariate imputation by chained equations in R. J Stat Softw.

[24] Thorpe LE, Greene C, Freeman A, Snell E, Rodriguez-Lopez JS, Frankel M (2015). Rationale, design and respondent characteristics of the 2013-2014 New York City health and nutrition examination survey (NYC HANES 2013-2014). Prev Med Rep.

[25] Lumley T (2004). Analysis of complex survey samples. J Stat Softw.

[26] Valliant R (1993). Poststratification and conditional variance estimation. J Am Stat Assoc.

[27] Chan PY, Zhao Y, Lim S, Perlman SE, McVeigh KH (2018). Using calibration to reduce measurement error in prevalence estimates based on electronic health records. Prev Chronic Dis.

[28] Raghunathan TE (2006). Combining information frommultiple surveys for assessing health disparities. Allg Stat Arch.

